# Cross-walking personality disorder types to ICD-11 trait domains: An overview of current findings

**DOI:** 10.3389/fpsyt.2023.1175425

**Published:** 2023-04-06

**Authors:** Jonatan Simon, Bastian Lambrecht, Bo Bach

**Affiliations:** ^1^Center for Personality Disorder Research (CPDR), Psychiatric Research Unit, Region Zealand, Slagelse, Denmark; ^2^Department of Psychology, University of Southern Denmark, Odense, Denmark

**Keywords:** ICD-11 (International Classification of Diseases), personality disorder (PD), personality trait, SCID-5-PD, ICD-10, dimensional, DSM-5 (the diagnostic and statistical manual of mental disorders), domain specifier

## Abstract

The ICD-11 has adopted a classification of Personality Disorders (PD) that abolishes the established categorical PD types in favor of global severity classification with specification of individual trait domains. To facilitate and guide this profound transition, an overview of current research on empirical associations between established PD types and ICD-11 trait domains seems warranted. We identified a total of 9 relevant studies from 2018 to 2022, which were based on both clinical and community samples from U.S., China, Brazil, Denmark, Spain, Korea, and Canada. The patterns of associations with ICD-11 trait domains were systematically synthesized and portrayed for each PD type. Findings overall showed expected and conceptually meaningful associations between categorical PD types and ICD-11 trait domains, with only few deviations. Based on these findings, we propose a cross-walk for translating categorical PD types into ICD-11 trait domains. More research is needed in order to further guide continuity and translation between ICD-10 and ICD-11 PD classification in mental healthcare, including facet-level ICD-11 trait information. Moreover, the nine reviewed studies only relied on self-reported ICD-11 trait domains, which should be expanded with clinician-rated trait domains in future research. Finally, future research should also take ICD-11’s essential PD severity classification into account.

## 1. Introduction

The newly released International Classification of Diseases 11th edition (ICD-11) (1) includes a fundamentally new approach to Personality Disorder (PD) diagnosis that relies on classification of global PD severity (i.e., Mild, Moderate, and Severe) and specification of one or more trait domains (i.e., Negative Affectivity, Detachment, Dissociality, Disinhibition, and Anankastia).^1^ Thus, the traditional PD types are abolished in favor of a new dimensional classification.

The ICD-11 trait domain specifiers may be used by the clinician to describe the most prominent individual characteristics of a patient’s personality that contribute to the personality disturbances (1). These trait domain specifiers can be considered homogenous building blocks of personality pathology, which may help disentangle and explain the overlapping or co-occurring features that exist across PD categories (2). Rather than abolishing stylistic features as we know them from the traditional PD typology, this new framework can be said to offer a more empirically sound stylistic framework. Thus, clinicians should still have the opportunity to characterize personality style, but now with a new palette of primary colors and flavors that may be blended in various ways (3). Different compositions of trait domains reflect different kinds of difficulty and may inform and guide specific approaches to understanding and treating the patient. For example, it makes a difference whether the PD is associated with the patient being overly anxious and avoidant (e.g., Negative affectivity and Detachment) or being excessively self-centered and reckless toward others (e.g., Dissociality and Disinhibition).

A similar approach has already been introduced 10 years ago in DSM-5’s Alternative Model for Personality Disorders (AMPD), which also allows clinicians to specify up to five trait domains (i.e., Negative Affectivity, Detachment, Antagonism, Disinhibition, and Psychoticism). The accumulating body of research on the AMPD trait domains is therefore helpful and informative when it comes to the preparation of the now official ICD-11 specification of trait domains (4, 5). Nevertheless, the two frameworks are not identical as the ICD-11 includes a separate domain of Anankastia partially corresponding to the opposite pole of Disinhibition, whereas the AMPD includes a separate domain of Psychoticism, which is not considered an aspect of PD by WHO.

A considerable number of publications have already addressed the trait-based conceptualization of PDs in general, primarily from the perspective of the AMPD criterion B traits (6, 7) and the Big Five model of normal traits (8–10), whereas only a small number of more recent studies have explicitly focused on the ICD-11 trait domains (5, 11, 12).

There are currently eight psychometrically sound approaches to the measurement of ICD-11 trait domains, which include the empirically based algorithm for the Personality Inventory for DSM-5 (PI-D) (13, 14), the Personality Inventory for ICD-11 (PiCD) (15), the Five-Factor inventory for ICD-11 (FFiCD) (16), The Personality Inventory for DSM-5 and ICD-11 Brief Form-Plus-Modified (PID5BF + M) (17, 18), the Informant Personality inventory for ICD-11 (IPiC) (19, 20), the Personality Assessment Questionnaire for ICD-11 personality traits (PAQ-11) (21), Clark et al.’s scales for ICD-11 Five Personality Disorder Trait Domains (22), and the Integrative Dimensional Personality Inventory-11 (IDPI-11) (23). Five of these measures (i.e., PID-5 algorithm, PiCD, FFiCD, PID5BF + M, and PAQ-11) are being employed in the studies reviewed in the present article.

### 1.1. The current review

In this short article, we aim to provide an overview of current research on the relationship between traditional PD types (i.e., Paranoid, Schizoid, Dissocial, Borderline, Histrionic, Anankastic, Anxious, Dependent, and Narcissistic) and ICD-11 trait domain specifiers (i.e., Negative affectivity, Detachment, Dissociality, Disinhibition, and Anankastia) by presenting and synthesizing findings from studies that explicitly operationalize all five ICD-11 trait domains. Subsequently, we discuss the identified pattern of associations for each PD type. Eventually, we propose how the synthesized findings may inform a “cross-walk” to be used by clinical practitioners in the transition from the traditional types to the new trait domain specifiers.

We used PubMed, PsycINFO, Web of Science, and a broad snowballing method to identify a total of nine relevant studies investigating associations between traditional PD types and ICD-11 trait domain scores (14, 17, 24–30). We chose to include exclusively articles published after 2017, with the rationale being that ICD-11 has gone through a number of iterations, in which diagnostic definitions have undergone significant changes (5, 31–34). The latest iteration of these was eventually settled in 2017, with the current established five trait domains (33).

## 2. Associations between personality disorder types and ICD-11 trait domains

Sampling, population, and measurement characteristics for each study are presented in Table 1 and bivariate associations are presented in Table 2. The studies included samples from both clinical and non-clinical populations across 7 countries. The trait domain scores were self-reported in all studies, whereas categorical PD types were based on clinical interviews in 3 studies and self-reports in 6 studies. Table 2 presents the bivariate correlations between PD types and ICD-11 trait domain scores for all nine studies, which we systematically summarize and discuss in the following for each PD type. We consistently focus on the two predominant trait domains for each PD type in terms of the magnitude of their correlation coefficients (see bolded coefficients in Table 2).

**Table 1:** Characteristics of seven studies reporting correlations between personality disorder types and ICD-11 trait domains.

Study	Sample (*N*)	Country	Measure of trait domains	Measure of PD types
Bach et al. 2018 (24)	Clinical (226)	Denmark	ICD-11 algorithm for PID-5	SCID-II
Lugo et al. 2019 (25)	Clinical (130) Community (656)	Brazil	ICD-11 algorithm for PID-5	Clinical diagnosis
Bach et al. 2020 (17)	Clinical (142)	Denmark	PID5BF + M	SCID-II
Sellbom et al. 2020 (14)	Clinical (343)	Canada	ICD-11 algorithm for PID-5	SCID-II-PQ
Kim et al. 2021 (21)	Clinical (75) At risk students (135)	Korea	PAQ-11	PBQ-SF
Fang et al. 2021 (27)	Students (3,550)	China	ICD-11 algorithm for PID-5	PDQ-4+
García et al. 2022 (28)	Community (758)	Spain	PiCD	IPDE
Sellbom et al. 2022 (29)	Community (428)	U.S.	PAQ-11	PDQ-4
Sorrel et al. 2022 (30)	Community (606)	Spain	FFiCD	IPDE

PID-5, Personality Inventory for DSM-5; SCID-II, Structured Clinical Interview for the DSM-IV-TR Axis II Disorders; SCID-II-PQ, SCID-II Personality Questionnaire; PID5BF + M, Personality Inventory for DSM-5 and ICD-11 Brief Form-Plus-Modified; PBQ-SF, Personality Belief Questionnaire-Short Form; PAQ-11, Personality Assessment Questionnaire ICD-11 version; PDQ-4−+, Personality Diagnostic Questionnaire 4 +; PiCD, Personality Inventory for ICD-11; IPDE, International Personality Disorder Examination; FFiCD, Five-Factor inventory for ICD-11.

**Table 2:** Associations between personality disorder types and ICD-11 trait domain specifiers across the nine identified studies.

ICD-10 PD types	ICD-11 trait domain specifiers				
	NA	DET	DISS	DIN	ANA
Paranoid PD					
Bach et al. (2018)	0.45**	0.43**	0.52**	0.48**	0.44**
Lugo et al. (2019)	n/a	n/a	n/a	n/a	n/a
Bach et al. (2020)	0.37**	0.29**	0.33**	0.23**	0.21*
Kim et al. (2020)	0.41**	0.46**	0.38**	0.33**	0.28**
Sellbom et al. (2020)	0.47**	0.26**	0.42**	0.32**	0.37**
Fang et al. (2021)	0.51**	0.21**	0.43**	0.21**	0.37**
Garcia et al. (2022)^a^	**0.36**	0.23	**0.37**	0.26	0.07
Sellbom et al. (2022)^a^	**0.47**	**0.37**	0.28	0.32	0.28
Sorrel et al. (2022)^a^	**0.41**	0.29	**0.42**	0.28	0.11
Schizoid PD					
Bach et al. (2018)	0.06	0.46**	0.31**	0.44**	0.40**
Lugo et al. (2019)	n/a	n/a	n/a	n/a	n/a
Bach et al. (2020)	−0.18*	0.28**	0.22**	0.06	0.05
Kim et al. (2020)	0.30**	0.44**	0.21**	0.26**	0.11
Sellbom et al. (2020)	0.15	0.51**	**0.19**	0.18	0.14
Fang et al. (2021)	0.29**	0.49**	0.09**	0.13**	0.24**
Garcia et al. (2022)^a^	0.08	**0.47**	0.05	−0.03	**0.11**
Sellbom et al. (2022)^a^	**0.36**	**0.52**	0.28	0.18	0.11
Sorrel et al. (2022)^a^	**0.15**	**0.52**	0.13	0.04	0.08
Dissocial PD					
Bach et al. (2018)	−0.09	0.26**	0.60**	0.49**	0.15
Lugo et al. (2019)	0.46*	0.42*	0.86*	0.84*	0.40*
Bach et al. (2020)	−0.36**	0.00	0.53**	0.33**	0.03
Kim et al. (2020)	0.38**	0.37**	0.39**	0.31**	0.26**
Sellbom et al. (2020)	0.08	0.01	0.29**	**0.17**	0.08
Fang et al. (2021)	0.28**	0.07**	0.33**	0.42**	0.14**
Garcia et al. (2022)^a^	0.18	0.04	**0.56**	**0.39**	−0.22
Sellbom et al. (2022)^a^	0.29	0.18	**0.36**	**0.48**	0.22
Sorrel et al. (2022)^a^	0.21	0.10	**0.56**	**0.40**	−0.21
Borderline PD					
Bach et al. (2018)	0.51**	0.38**	0.43**	0.60**	0.48**
Lugo et al. (2019)	0.88*	0.46*	0.69*	0.77*	0.61*
Bach et al. (2020)	0.45**	0.25**	0.25**	0.44**	0.25**
Kim et al. (2020)	0.58**	0.43**	0.24**	0.35**	0.15*
Sellbom et al. (2020)	0.61**	0.19	0.42**	0.52**	0.40**
Fang et al. (2021)	0.65**	0.29**	0.34**	0.47**	0.41**
Garcia et al. (2022)^a^	**0.60**	0.09	0.29	**0.52**	−0.16
Sellbom et al. (2022)^a^	**0.60**	0.35	0.31	**0.45**	0.31
Sorrel et al. (2022)^a^	**0.62**	0.26	0.52	**0.60**	−0.07
Histrionic PD					
Bach et al. (2018)	0.29**	0.04	0.32**	0.43**	0.34**
Lugo et al. (2019)	n/a	n/a	n/a	n/a	n/a
Bach et al. (2020)	0.26**	−0.04	0.36**	0.36**	0.22**
Kim et al. (2020)	0.40**	0.16*	0.31**	0.37**	0.07
Sellbom et al. (2020)	0.06	−0.25**	0.34**	0.23	0.03
Fang et al. (2021)	0.36**	−0.02	0.45**	0.31**	0.29**
Garcia et al. (2022)^a^	**0.35**	−0.19	0.28	**0.39**	−0.16
Sellbom et al. (2022)^a^	**0.36**	−0.01	0.19	**0.37**	0.39
Sorrel et al. (2022)^a^	0.32	−0.04	**0.42**	**0.39**	−09
Anankastic PD					
Bach et al. (2018)	0.23*	0.15	0.26**	0.13	0.62**
Lugo et al. (2019)	0.61*	0.78*	0.59*	0.43*	0.89*
Bach et al. (2020)	0.26**	0.09	0.17	0.04	0.66**
Kim et al. (2020)	0.28**	0.30**	0.25**	0.19**	0.47**
Sellbom et al. (2020)	0.25**	0.14	0.18	0.12	0.54**
Fang et al. (2021)	0.43**	0.32**	0.28**	−0.19**	0.53**
Garcia et al. (2022)^a^	**0.37**	0.20	0.19	0.03	**0.35**
Sellbom et al. (2022)^a^	**0.40**	0.24	0.29	0.32	**0.33**
Sorrel et al. (2022)^a^	**0.33**	0.29	0.29	0.01	**0.50**
Anxious (Avoidant) PD					
Bach et al. (2018)	0.54**	0.33**	0.00	0.18*	0.35**
Lugo et al. (2019)	0.78*	0.83*	0.53*	0.43*	0.61*
Bach et al. (2020)	0.50**	0.33**	−0.11	0.09	0.21*
Kim et al. (2020)	0.51**	0.42**	0.25**	0.38**	0.16*
Sellbom et al. (2020)	0.53**	0.53**	0.13	0.28**	0.31**
Fang et al. (2021)	0.53**	0.35**	0.23**	0.32**	0.38**
Garcia et al. (2022)^a^	**0.46**	**0.49**	0.11	0.15	0.26
Sellbom et al. (2022)^a^	**0.60**	**0.35**	0.27	0.35	0.20
Sorrel et al. (2022)^a^	**0.53**	**0.52**	0.22	0.20	0.29
Dependent PD					
Bach et al. (2018)	0.46**	0.17*	0.06	0.34**	0.24**
Lugo et al. (2019)	n/a	n/a	n/a	n/a	n/a
Bach et al. (2020)	0.47**	0.16	−0.01	0.30**	0.05
Kim et al. (2020)	0.47**	0.21**	0.21**	0.38**	0.03
Sellbom et al. (2020)	0.40**	0.10	−0.02	0.29**	0.32**
Fang et al. (2021)	0.47**	0.19**	0.26**	0.35**	0.37**
Garcia et al. (2022)^a^	**0.43**	0.10	0.08	**0.27**	0.09
Sellbom et al. (2022)^a^	**0.53**	0.17	0.26	**0.41**	0.22
Sorrel et al. (2022)^a^	**0.48**	0.25	0.25	**0.36**	0.03
Narcissistic PD					
Bach et al. (2018)	0.09	0.13	0.67**	0.36**	0.43**
Lugo et al. (2019)	0.41*	0.28*	0.68*	0.43*	0.49*
Bach et al. (2020)	−0.11	−0.01	0.67**	0.27**	0.12
Kim et al. (2020)	0.17*	0.24**	0.13	0.26**	0.34**
Sellbom et al. (2020)	0.27*	0.19**	0.65**	0.29**	0.22**
Fang et al. (2021)	0.50**	0.21**	0.50**	0.32**	0.41**
Garcia et al. (2022)^a^	0.12	0.01	**0.49**	**0.21**	0.04
Sellbom et al. (2022)^a^	0.37	0.20	0.33	**0.40**	**0.40**
Sorrel et al. (2022)^a^	0.14	0.03	**0.54**	**0.18**	0.05

NA, Negative affectivity; DET, Detachment; DISS, Dissociality; DIN, Disinhibition; ANA, Anankastia.

*p < 0.05; **p < 0.001.

a Statistical significance not reported. The two most predominant trait domains for each PD type, in terms of the magnitude of their correlation coefficients, are bolded.

Lugo et al. (25) reported Spearman’s ρ coefficients and they only investigated the six PD types that correspond to the AMPD hybrid types, including schizotypal PD, and coefficients for certain PD types are therefore not reported.

### 2.1. Paranoid

Paranoid PD was primarily associated with the trait domains of Negative affectivity and Dissociality, in that order. The primary role of Negative affectivity seems conceptually meaningful because mistrustfulness is a core feature of Paranoid PD as well as an explicit feature of ICD-11’s definition of Negative Affectivity. The secondary role of Dissociality is consistent with previous research and empirical frameworks of psychopathology suggesting that features of Paranoid PD belong to the spectrum of externalizing disorders (35, 36). Moreover, the Paranoid PD type is characterized by a combative and tenacious sense of self-righteousness and a tendency to experience excessive self-aggrandizing (37), which is somewhat indicative of features defining the Dissociality domain such as anger, temper tantrums, and denigration of others combined with certain aspects of self-centeredness (1). Three studies also showed substantial associations with Detachment (21, 24, 29), which is also consistent with previous research (7) and conceptualizations (38).

### 2.2. Schizoid

Schizoid PD was consistently associated with the trait domain of Detachment, which is explicitly defined by features of social detachment including limited capacity for enjoyment and lack of social interactions and intimate relationships along with emotional detachment including aloofness with limited emotional experience and expression (1). This description is substantially consistent with the ICD-10 definition of Schizoid PD, which includes a limited capacity to express feelings and to experience pleasure as well as withdrawal from affectional, social, and other contacts (37).

### 2.3. Dissocial (antisocial)

Dissocial PD was consistently associated with the trait domain of Dissociality and Disinhibition, in that order. This is consistent with meta-analytic evidence indicating that Dissocial PD is characterized by both antagonistic features of callousness and lack of remorse as well as disinhibited features of recklessness, risk taking, and impulsivity (6, 7). In other words, the established Dissocial/Antisocial PD is actually a combination of Dissociality and Disinhibition, and not a pure expression of dissociality or antagonism. With the ICD-11 trait domain specifiers, clinicians are allowed to code a more pure expression of features corresponding to psychopathy including features such as lack of empathy and grandiosity. Moreover, based on a clinical interview-rated sample, Bach et al. (24) also found Negative affectivity to be negatively correlated with Dissociality, which may indicate expected features of stress-immunity, boldness, and fearlessness that often characterize such individuals (39).

### 2.4. Emotionally unstable (borderline)

Borderline PD was almost consistently and primarily associated with high scores on Negative affectivity and Disinhibition, which aligns with the fact that this PD type is essentially characterized by emotion dysregulation (i.e., Negative affectivity) and self-destructive impulsivity (i.e., Disinhibition). As evident from Table 2, there is a broad pattern of substantial correlations with Borderline PD, beyond Negative affectivity and Disinhibition, which underscores the heterogeneity and “catch-all” features of this PD category (40–43). In addition to the nine included studies, other studies also support that the Borderline pattern is primarily associated with PiCD, PID5BF + M, and clinician-rated scores of Negative affectivity and Disinhibition, in that order (40, 44–47).

### 2.5. Histrionic

Histrionic PD showed a mixed pattern of small to moderate associations with Dissociality, Disinhibition, and Negative Affectivity, which aligns with the fact that this PD type is essentially characterized by self-centeredness and longing for attention (i.e., Dissociality), excitement and attention seeking (i.e., Disinhibition), and excessive and labile emotionality (i.e., Negative Affectivity). Two studies also indicated negative associations with Detachment (14, 28), which is consistent with the extreme extraversion and emotional expressivity (e.g., reversed Detachment) characterizing Histrionic PD.

### 2.6. Anankastic (obsessive–compulsive)

Anankastic PD was consistently associated with the trait domain of Anankastia and secondarily with Negative Affectivity, which aligns with the fact that this PD type is characterized by aspects of both perfectionism (e.g., pedantry, rigidity, and extreme orderliness) and behavioral constraint (e.g., risk aversion) as well as some feelings of excessive doubt and caution (i.e., Negative affectivity). Interestingly, based on a clinical interview-rated sample, Bach et al. (24) also found the trait domain of Dissociality to be somewhat associated with Anankastic PD, which may indicate features related to unreasonable insistence that others submit to exactly their way of doing things. This is consistent with research showing that Anankastic PD features are partially associated with aggression (48) and hostile-dominant interpersonal problems (49). Moreover, Lugo et al. (25) found Detachment to characterize this PD type, which may be attributed to the anankastic features of exclusion of pleasure and interpersonal relationships in favor of productivity.

### 2.7. Anxious (avoidant)

Anxious PD was consistently associated with the trait domains of Negative affectivity and Detachment, which aligns with the fact that this PD type is essentially characterized by anxiousness and low self-esteem exhibited as avoidance of situations and activities (i.e., Negative Affectivity) along with interpersonal and social withdrawal (i.e., Detachment). Moreover, the majority of the studies also showed substantial associations with Anankastia, which may indicate the emotional constraint and overconcern about avoiding potential negative consequences of any activity characterizing individuals with Avoidant PD (50, 51).

### 2.8. Dependent

Dependent PD was consistently associated with Negative Affectivity, which aligns with the fact that this PD type is essentially characterized by low self-confidence exhibited as dependency and frequent reliance on others for advice, direction, and other kinds of help. Moreover, and perhaps surprisingly, the majority of studies also showed substantial associations with Disinhibition. This secondary pattern may be attributed to ICD-11’s inclusion of irresponsibility (or lack of desire to take responsibility) for defining Disinhibition, which is also consistent with previous PID-5 research on Dependent PD (52–54). Moreover, expert literature also suggests that impulsivity may be naturally associated with trait dependency (55).

### 2.9. Narcissistic

Narcissistic PD was almost consistently associated with the trait domain of Dissociality, and secondarily with both Anankastia and Disinhibition. The primary association with Dissociality aligns with the self-centeredness, entitlement, expectation of others’ admiration, and lack of empathy defining this domain. The association with Anankastia may indicate “narcissistic perfectionism,” which serves to enhance competitiveness, self-esteem, and grandiose self-presentation (56). The association with Disinhibition may indicate a tendency to overestimate own abilities (i.e., recklessness), difficulty delaying reward and satisfaction due to a sense of entitlement (i.e., impulsivity), and a narcissistic pattern of procrastination instead of making a realistic plan for their lives (i.e., irresponsibility and lack of planning) (57–59).

## 3. Discussion

The field is gradually leaving the categorical PD types behind in favor of a new empirically informed approach that is now officially introduced by WHO in the ICD-11 (1). However, the transition from the familiar types to a fundamentally new framework may be challenging for many old residents in mental healthcare. We therefore set out to present the first overview of associations between traditional PD types and the new ICD-11 trait domain specifiers. It is important to underscore that such empirical associations should not be considered evidence for criterion or construct validity because the PD types do not comprise scientifically sound criterion measures. In fact, the psychometric shortcomings of the traditional PD categories comprise a major reason for exchanging them with a new classification (60, 61). Therefore, the associations should only be considered indications of continuity and translatability of historically important stylistic features.

### 3.1. A cross-walk where stylistic features are not lost in translation

The identified pattern of associations was overall found to be conceptually meaningful and consistent with previous research and theoretical propositions (e.g., meta-analytic evidence from research on the Five-Factor Model and the AMPD trait model) (6–9, 62). Thus, the presented pattern of associations may guide and inform clinical practitioners with respect to the translation from the familiar PD types to the new stylistic features of trait domains. Even though the traditional PD types are abolished, their stylistic features do not seem to be lost in translation. Based on findings in the present overview, we have proposed a clinician-friendly cross-walk as shown in Supplementary Table S1.

### 3.2. The significance of Anankastia

In contrast to DSM-5’s AMPD framework, the ICD-11 classification includes a separate domain of Anankastia corresponding to Compulsivity and partially to reversed Disinhibition. In the present overview, we found that the trait domain of Anankastia accounts for essential features of Anankastic (obsessive–compulsive) PD, as expected, while it somewhat also accounts for features of Narcissistic PD (e.g., narcissistic perfectionism) and Avoidant PD (e.g., risk aversion and overconcern). Negative associations with Disinhibition (i.e., reversed Disinhibition) did not seem to account for these features, which supports WHO’s decision of including a separate domain of Anankastia. For example, Narcissistic PD was characterized by both Disinhibition (e.g., entitlement expressed as difficulty delaying reward and satisfaction) and Anankastia (e.g., narcissistic perfectionism, vanity, and control), which would not be possible to portray and code simultaneously on a single bipolar domain of Disinhibition (i.e., low versus high Disinhibition). This is overall consistent with empirical findings and clinical arguments supporting the utility of a separate domain of Anankastia (17, 63–66), while recognizing that this domain is substantially but not entirely the polar opposite of Disinhibition (15, 20, 67).

### 3.3. The complexity of borderline and narcissism

Two of the most indistinct and heterogeneous PD types across the nine studies were Borderline PD and Narcissistic PD, which both seem to allow for different expressions and trait constellations.

Borderline PD was captured by a broad pattern of trait domains ranging from internalizing features (e.g., Negative affectivity) to externalizing features (i.e., Disinhibition). This composition seems consistent with research suggesting that Borderline is not a distinct PD type but rather an index of global personality pathology and severity, which aligns with the original metaphorical use of the term “borderline” or “borderland” (43, 68). The substantial but mixed associations with the other three trait domains also underscore the “catch all” features of this syndrome (69). It therefore seems reasonable if the borderline pattern serves as a transitional specifier that eventually is phased out in the coming era (40, 47).

Narcissistic PD is another PD type that is not straight forward to characterize using trait domains, which also seems related to the many possible faces of narcissism. It makes a substantial difference whether narcissistic PD is characterized by vulnerable features (e.g., Negative affectivity), perfectionistic-controlling features (e.g., Anankastia) or features of impatience and self-stimulating impulses due to a sense of entitlement (e.g., Disinhibition). More broadly, the role of Disinhibition may also indicate aspects of procrastination (i.e., lack of planning and goal-directedness) as often seen in vulnerable narcissism. Overall, the complex constellation of trait domains for narcissistic features is consistent with the traditional conceptualization that Narcissistic PD involves moderate–severe impairments in personality functioning (70, 71).

### 3.4. Limitations and future directions

The findings presented in this review should be considered in the light of several potential limitations. First, due to the scarcity of identified studies, we could not perform a meta-analysis in order to produce a quantitative analytical synthesis of the data but pursued to conduct a scoping review instead with less restrictive criteria (72). Third, the methods and instruments used to assess or operationalize the PD types and ICD-11 trait domains varied significantly, which may explain certain deviations and inconsistencies in the findings. For example, Kim et al. (26) used the Personality Belief Questionnaire-Short Form (PBQ-SF) to measure features of the corresponding PD types, while Lugo et al. (25) used clinical diagnoses of PD types with no standardized instrument. The coefficients reported in Lugo et al. (25) were remarkably larger than coefficients reported in the other studies, which may be attributed to the use of Spearman’s *ρ* rather than Pearson’s *r*. Nevertheless, the pattern of their findings was largely consistent with findings in the other studies, while particular deviations may also be attributed to differing operationalizations. Fourth, future research should integrate clinician-ratings of ICD-11 trait domains to account for issues such as mono-method bias (19, 20). Fifth, future studies (and reviews) should also include facet-level information for each trait domain, which may provide a more sophisticated portrayal of the continuity (e.g., FFiCD facets and nuances of grandiosity and vanity may do a better job at capturing Narcissistic PD). Sixth, future reviews might also seek to include studies that investigate the ability of ICD-11 trait domains to differentiate established PD diagnoses and other diagnostic categories (25, 63, 73–75), which may also highlight certain aspects of diagnostic continuity. Finally, the ICD-11 PD diagnosis first and foremost relies on severity classification (i.e., mild, moderate, and severe), which was not taken into account in this review due to insufficient published research. We therefore suggest that a future overview article seeks to synthesize how familiar PD types are best portrayed according to PD severity (76–78).

## Author contributions

BB conceptualized the idea, supervised during the writing process, and provided critical revisions. BL and JS conducted the systematic search and prepared the first draft of the manuscript. All authors contributed to the article and approved the submitted version.

## Conflict of interest

The authors declare that the research was conducted in the absence of any commercial or financial relationships that could be construed as a potential conflict of interest.

## Publisher’s note

All claims expressed in this article are solely those of the authors and do not necessarily represent those of their affiliated organizations, or those of the publisher, the editors and the reviewers. Any product that may be evaluated in this article, or claim that may be made by its manufacturer, is not guaranteed or endorsed by the publisher.

## Footnotes

^1^After classification of PD severity and specification of trait domains, the ICD-11 also offers clinicians the opportunity to specify a borderline pattern, which was included for pragmatic reasons to facilitate some continuity with established clinical practice.
