# Supplementary material for: Cross-walking personality disorder types to ICD-11 trait domains: An overview of current findings
Source: Front Psychiatry. 2023 Apr 6;14:1175425. doi: 10.3389/fpsyt.2023.1175425 (PMC10116048; doi:10.3389/fpsyt.2023.1175425)
Supplement: Supplementary file 1 [file Data_Sheet_1.PDF]

Supplementary Material

Cross-Walking Personality Disorder Types to ICD-11 Trait Domains: An Overview of Current Findings

Jonatan Simon [1,2†], Bastian Lambrecht [1,2†], Bo Bach [1,2*]
[1]Center for Personality Disorder Research (CPDR), Psychiatric Research Unit, Region Zealand, Denmark
[2]Department of Psychology, University of Southern Denmark
[†]Shared authorship.

*** Correspondence:**
Bo Bach, PhD; bbpn@regionsjaelland.dk

Supplementary Table S1.

Tentative Cross-walk matrix for translating Personality Disorder types into prominent trait domains

	Negative affectivity	Detachment	Dissociality	Disinhibition	Anankastia
**Paranoid**	+	+	+		
**Schizoid**		++			
**Dissocial**	-		++	++	
**Borderline**	++		+	++	
**Histrionic**	+	-	+	+	
**Anankastic**	+			-	++
**Avoidant**	++	++			+
**Dependent**	++			+[a]	
**Narcissistic**	+		++	+	+

Note. The symbols indicate strongly prominent (++), prominent (+), and absent/reversed (-). [a]mainly features of irresponsibility. The pattern is derived from the most prominent correlations for each PD type (see Table 2).
